# Hypericin-photodynamic therapy leads to interleukin-6 secretion by HepG2 cells and their apoptosis via recruitment of BH3 interacting-domain death agonist and caspases

**DOI:** 10.1038/cddis.2013.219

**Published:** 2013-06-27

**Authors:** M Barathan, V Mariappan, E M Shankar, B JJ Abdullah, K L Goh, J Vadivelu

**Affiliations:** 1 Tropical Infectious Disease Research and Education Center (TIDREC), Department of Medical Microbiology, University of Malaya, 50603 Kuala Lumpur, Malaysia; 2 Faculty of Medicine, Department of Biomedical Imaging, University of Malaya, 50603 Kuala Lumpur, Malaysia; 3 Faculty of Medicine, Department of Medicine, University of Malaya, Lembah Pantai, 50603 Kuala Lumpur, Malaysia

**Keywords:** apoptosis, caspase, HepG2, hypericin, IL-6, photodynamic therapy

## Abstract

Photodynamic therapy (PDT) has emerged as a capable therapeutic modality for the treatment of cancer. PDT is a targeted cancer therapy that reportedly leads to tumor cell apoptosis and/or necrosis by facilitating the secretion of certain pro-inflammatory cytokines and expression of multiple apoptotic mediators in the tumor microenvironment. In addition, PDT also triggers oxidative stress that directs tumor cell killing and activation of inflammatory responses. However, the cellular and molecular mechanisms underlying the role of PDT in facilitating tumor cell apoptosis remain ambiguous. Here, we investigated the ability of PDT in association with hypericin (HY) to induce tumor cell apoptosis by facilitating the induction of reactive oxygen species (ROS) and secretion of Th1/Th2/Th17 cytokines in human hepatocellular liver carcinoma cell line (HepG2) cells. To discover if any apoptotic mediators were implicated in the enhancement of cell death of HY-PDT-treated tumor cells, selected gene profiling in response to HY-PDT treatment was implemented. Experimental results showed that interleukin (IL)-6 was significantly increased in all HY-PDT-treated cells, especially in 1 *μ*g/ml HY-PDT, resulting in cell death. In addition, quantitative real-time PCR analysis revealed that the expression of apoptotic genes, such as BH3-interacting-domain death agonist (*BID*), cytochrome complex (*CYT-C*) and caspases (*CASP3, 6, 7, 8* and *9*) was remarkably higher in HY-PDT-treated HepG2 cells than the untreated HepG2 cells, entailing that tumor destruction of immune-mediated cell death occurs only in PDT-treated tumor cells. Hence, we showed that HY-PDT treatment induces apoptosis in HepG2 cells by facilitating cytotoxic ROS, and potentially recruits IL-6 and apoptosis mediators, providing additional hints for the existence of alternative mechanisms of anti-tumor immunity in hepatocellular carcinoma, which contribute to long-term suppression of tumor growth following PDT.

Hepatocellular carcinoma (HCC) is the most common liver cancer and the sixth most frequent neoplasms afflicting humankind.^1^ HCC is reportedly responsible for >600 000 deaths annually.^1^ Also known as adult primary liver cancer, HCC develops from an abnormal mass of tumor nodule and metastases to the adjoining parts of the liver eventually culminating in malignancy.^2, 3^ Accumulating lines of evidence suggest that HCC causes more than half a million new cases yearly.^3^ Progression to HCC is often attributed to the lack of pathognomonic symptoms and poor prognosis.^4, 5^ The currently available clinical interventions require extensive prior immunosuppressive treatments often leading to undesirable physiological events.^4^ Hence, recent advances in immunotherapy advocate measures to stimulate the immune system, especially the adaptive immune responses that could have a paramount role in tumor destruction/regression and, as such, could serve as promising selective therapeutic targets in cancer.^5^ Hence, the importance of novel anti-tumor therapeutic strategies with lesser adverse effects on patients remains an urgent need in clinical practice.

Photodynamic therapy (PDT) is a widely known effective therapeutic approach that requires a non-toxic photosensitizer (PS) drug and oxygen (O_2_) in concert with a harmless light source to facilitate selective destruction of tumorigenic cells.^6^ A plethora of PS drugs, for instance, porfimer sodium (Photofrin II)^7^ and 5-aminolevulinic acid,^8^ have been widely used in PDT. However, hypericin (HY), an extract from *Hypericum perforatum,* has recently been shown to be a selective anti-tumor PS agent with high-quantum yields and a low cytotoxicity.^9^ Several *in vitro* and *in vivo* investigations have established its anticancer potentials in conjunction with light irradiation. Previously published findings have confirmed the role of HY-PDT against tumor cell proliferation.^10^ Besides, HY has also been tested in numerous experimental therapeutics in concert with PDT on a myriad cancers and cell line experiments.^11^

Inflammatory responses induced by reactive oxygen species (ROS) is believed to be the key priming event in the development of anti-tumor immunity.^12^ The phototoxic reaction following HY-PDT initiates the release of proinflammatory mediators by triggering the release of interleukin (IL)-1*α*, IL-1*β*, Interferon (IFN)-*γ*, IL-6, tumor necrosis factor (TNF)-*α* and certain other chemokines that provoke a strong inflammatory response in PDT-treated tumor cells.^13^ Of note, IL-6, a pleiotropic cytokine implicated with barrier functions, is reported to trigger Th17 expansion. Furthermore, it is believed to have a paramount role in antitumor immunity at the site of inflammation owing to its neutrophil-mobilizing functions.^14^ Hence, IL-6 synthesized following PDT is believed to mediate antitumor responses, providing additional secondary mechanisms of PDT-induced tumor cell killing.

Despite these promising observations, clinical issues such as safe dosage of PS drugs and suitable light source that induce potential antitumor immunity remain to be addressed.^4^ With this backdrop of rationale, we have reasoned that proinflammatory cytokine mobilization and their recruitment by tumor cells could be increased in PDT-treated cells, leading to increased activation of immune responses against tumor progression via inflammation.^10^ Further, although the events triggering the antitumor functions of HY-PDT have been established *in vitro* against certain tumor models,^15^ the mechanisms underlying this effect have seldom been investigated. Here, we have shown that photo-oxidative (due to ROS induction) tumor cells and the eventual upregulation of IL-6-facilitated tumor cell death have underpinned the association of certain primary apoptotic mediators with inhibition of tumor growth. Furthermore, we have also established that IL-6 was consistently upregulated in PDT-treated cells, and their levels were associated with increased tumor cell apoptosis and caspase activities. We also evaluated the potential interaction between proinflammatory cytokines in the tumor microenvironment and the activation of apoptotic caspases in the presence of cytochrome complex (CYT-C) and BH3-interacting-domain death agonist (BID), pro-apoptotic factor in human hepatocellular liver carcinoma cell line (HepG2) cells following HY-PDT treatment.

## Results

### HY-PDT inhibits survival of HepG2 cells with morphological changes identical to apoptosis

**Figure 1:**
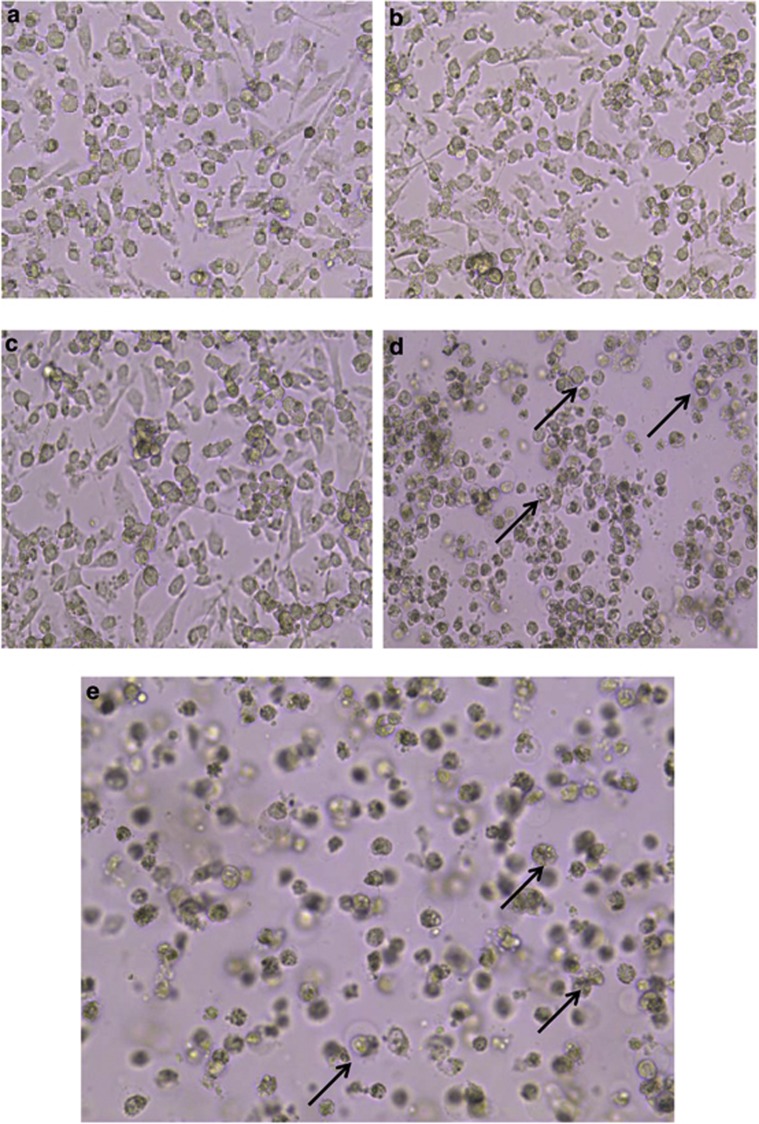
HY-PDT apoptotic effects on cellular morphology of HepG2 human hepatocellular carcinoma cells. Cells were treated with increasing concentrations of HY (0.1, 0.2, 0.5 and 1 *μ*g/ml HY) for 8 h followed by irradiation for 5 min. The cells were further incubated for 18 h and cellular morphology was examined for each treatment. (**a**) Cellular morphology of untreated HepG2 cells. The cells were aggregated and clustered as monolayer forms. (**b** and **c**) Early morphological changes during apoptosis in 0.1 and 0.2 *μ*g/ml HY-PDT-treated cells, respectively. Cells were elongated and reduction of cell growth was observed. (**d** and **e**) Late apoptotic morphological changes in cells induced by 0.5 and 1 *μ*g/ml HY-PDT compared with mock untreated cells. The cell confluency appeared to reduce from ∼90% in untreated to ∼20% in 1 *μ*g/ml HY-treated cells. Apoptotic cells were detected as cell shrinkage and membrane blebbing. Similar cellular morphology was observed in three independent experiments (magnification × 100). The half-headed arrows represent apoptotic cells

To qualitatively test whether increasing concentrations of HY in PDT treatment could inhibit survival of HepG2 cells, we examined the morphological changes brought in by apoptosis following HY-PDT treatment using inverted light microscopy. Large spherical cells that eventually assumed clumped and/or aggregate forms were observed in the untreated cells (Figure 1a). In contrast, 0.1 and 0.2 *μ*g/ml of HY-PDT-treated cells did not show any visible signs of cell death (Figures 1b and c). However, the 0.5 and 1 *μ*g/ml HY-treated cells showed distinctive and prominent morphological signs of apoptosis (Figures 1d and e), that is, the cells eventually assumed small, isolated or detached forms, showing characteristic signs of blebbing and shrinking of the cell membrane (Figures 1d and e). The untreated HepG2 cells formed highly confluent cell monolayers as compared with the 0.5 and 1 *μ*g/ml HY-treated cells, and frequently showed floating dead cells, which is believed to be attributed to the formation of membrane-bound apoptotic bodies, reduced cell volume and chromatin condensation.^16^ Consistently, these results showed that 0.5 and 1 *μ*g/ml of HY were capable of inducing cell death in HY-PDT-treated HepG2 cells with morphological changes that were identical to programmed cell death.

**Figure 2:**
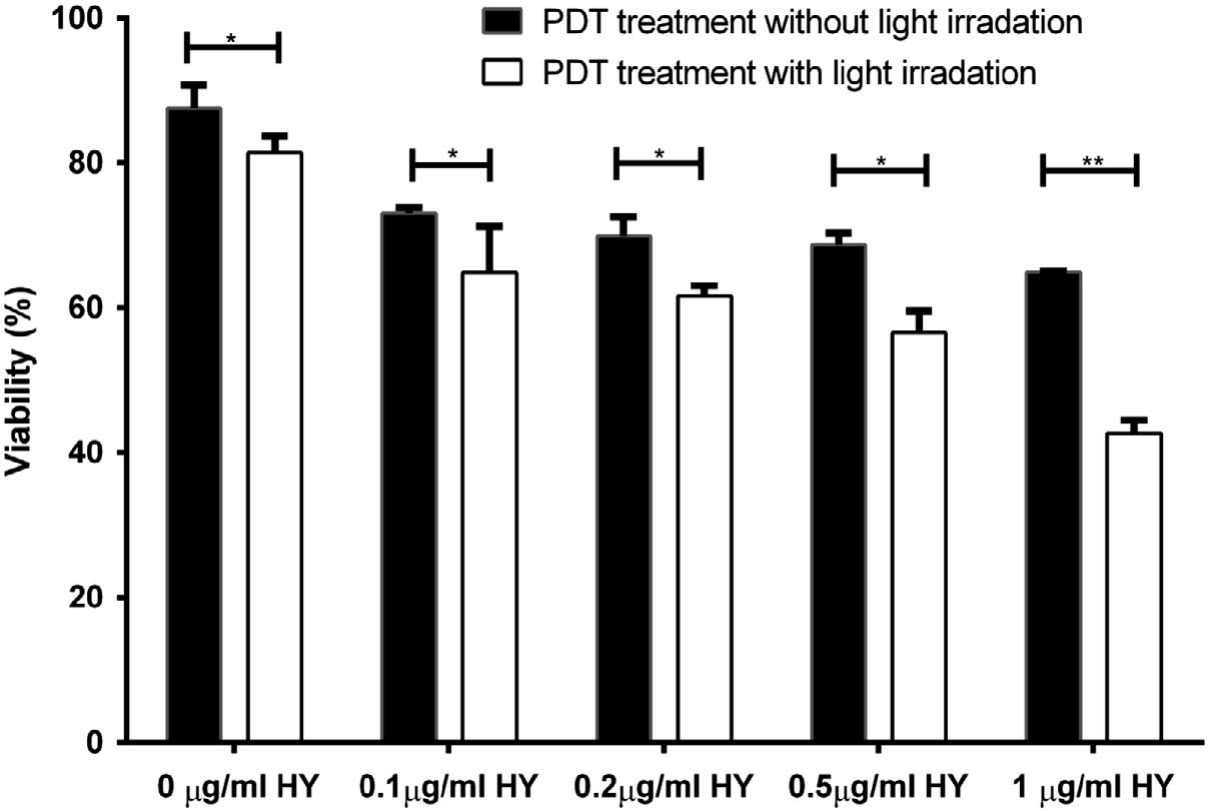
The effect of light irradiation in HY-PDT reduces viability of HepG2 cells. Cell viability profile for HepG2 cells exposed to increasing amounts of HY with or without light irradiation. All cells were treated with 0, 0.1, 0.2, 0.5 or 1 *μ*g/ml of HY for 8 h with or without light irradiation and incubated for 24 h in the dark. PrestoBlue cell viability assay was performed after 24 h incubation. The percentage of viable cells exposed to various doses of HY was normalized against that of untreated cells. Data are mean±S.E.M. of *n*=3 independent experiments carried out in triplicates. Statistically significant differences are labeled as *P<0.05 and **P<0.01, compared with non-irradiated cells using Student's unpaired two-tailed *t*-test

Now, to quantitatively test whether HY concentration was important in PDT treatment for inhibiting cell proliferation, we compared the photocytotoxicity of HepG2 cells treated with increasing concentrations of HY using a PrestoBlue cell viability assay. As shown in Figure 2, the viability of HepG2 cells treated with HY-PDT and HY alone declined in a dose-dependent manner illustrating a similar cytotoxic trend. However, we observed that the rate of proliferation of cells exposed to HY and light was significantly much lower than the HY-treated cells without light exposure. The proliferative rates of treated HepG2 cells with light were 81%, 69%, 61%, 55% and 40%, with concentrations ranging between 0, 0.1, 0.2, 0.5 and 1 *μ*g/ml, respectively (Figure 2). In contrast, the proliferative rates of non-irradiated cells were 91%, 86%, 76.2%, 75% and 71% with concentrations ranging between 0, 0.1, 0.2, 0.5 and 1 *μ*g/ml, respectively (Figure 2). Of note, the inhibitory effect was stronger with the different HY concentrations used, with the most significant effect observed in the 1 *μ*g/ml-treated cells. Taken together, our data confirm that irradiated HY in PDT treatment is necessary to effectively inhibit tumor cell proliferation.

### HY-PDT triggers HepG2 cell death via mechanisms consistent to apoptosis

**Figure 3:**
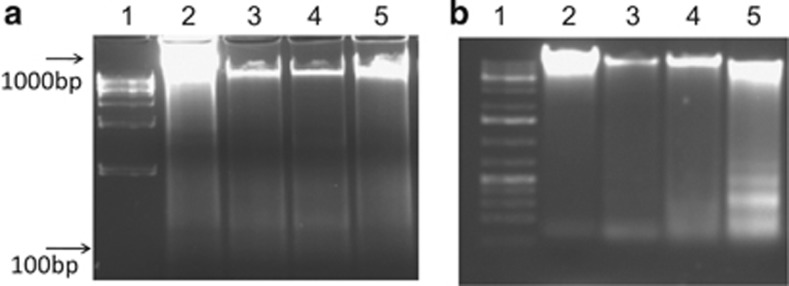
The effect of light irradiation in HY-PDT induces DNA fragmentation indicating apoptosis. HepG2 cells were treated with HY at increasing concentrations followed by no light irradiation (**a**) or with light irradiation for 5 min (**b**) and further incubation for 18 h. (**a** and **b**) Lane 1: 1 kb DNA ladder; lane 2: untreated cells; lane 3: cells treated with 0.2 *μ*g/ml HY; lane 4: cells treated with 0.5 *μ*g/ml HY and lane 5: cells treated with 1 *μ*g/ml HY. No obvious DNA laddering was seen in the gel. An obvious DNA laddering was formed in the cells treated with 1 *μ*g/ml HY-PDT. Nucleosomal DNA was fragmented into oligonucleosomes (sized 180–200 bp) in 1 *μ*g/ml HY-PDT-treated cells

**Figure 4:**
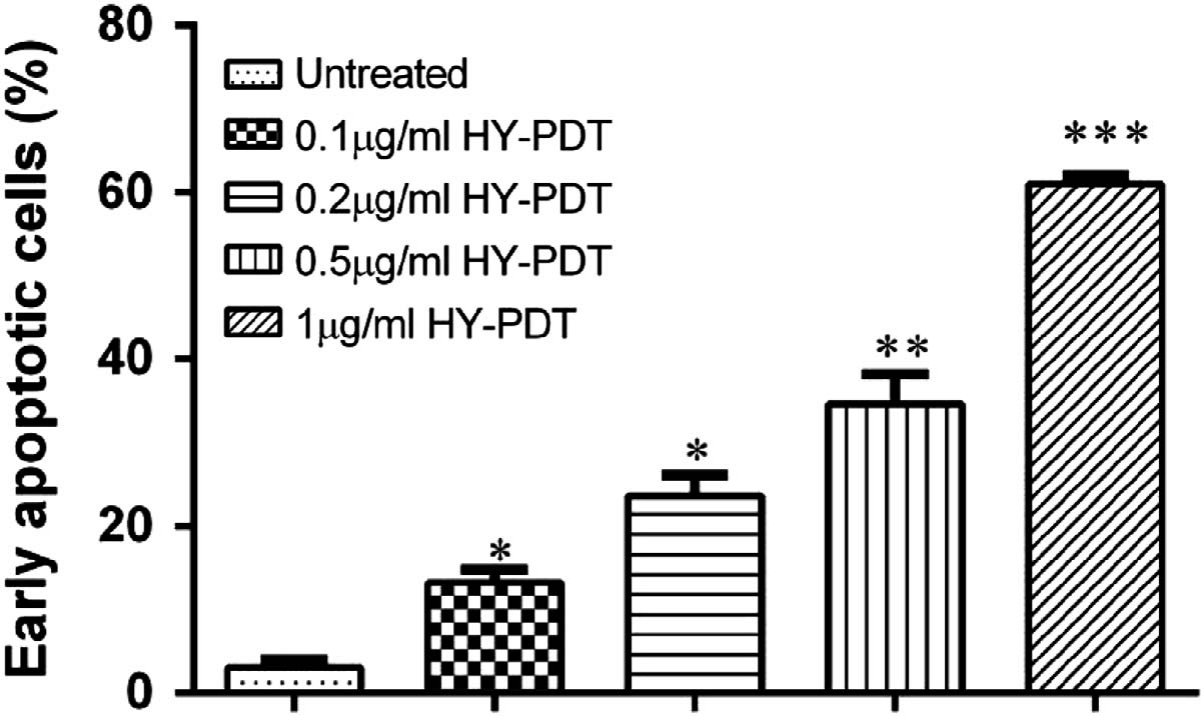
HY-PDT induces apoptosis in HepG2 cells. The cells were treated with 0.1, 0.2, 0.5 and 1 *μ*g/ml HY for 8 h and irradiated for 5 min. The rate of apoptosis of treated cells was determined 18 h post incubation. The percentage of apoptotic cells was determined by FACSCanto II and analyzed using a FCAP array (BD Biosciences). The apoptotic rate of HepG2 cells was concentration dependent after HY-PDT exposure. Data are mean±S.E.M. of *n*=3 independent experiments carried out in triplicates. Statistically significant differences are labeled as *P<0.05, **P<0.01 and ***P<0.001, compared with untreated cells using Student's unpaired two-tailed *t*-test

To investigate the potential mechanism that recruits HepG2 cell death following PDT, we next set out to decipher the effect of PDT in contributing to target cell apoptosis by looking for DNA fragmentation and annexin V-fluorescein isothiocyanate (FITC)/propidium iodide (PI)-stained cells using flow cytometry. Treatment without light irradiation at increasing concentrations of HY did not cause nucleosomal DNA fragmentation (Figure 3a). A similar result was observed for cells treated with light irradiation at concentrations 0.2 and 0.5 *μ*g/ml of HY (Figure 3b). However, there was a major increase in DNA laddering (fragmentation of oligonucleosomal size between180 bp and 200 bp) after treatment with light irradiation at 1 *μ*g/ml of HY concentration. As DNA fragmentation does not differentiate between apoptotic and necrotic cell death, the induction of early and late apoptotic cells was verified by measuring the detection of externalization of membrane phospholipid phosphatidylserine and the loss of membrane integrity using flow cytometry. HY-PDT caused a concentration-dependent increase in the percentage of HepG2 cells that were early apoptotic (annexin V-FITC^+^/PI^−^; *P*<0.001; Figure 4). This convincingly showed that the 1 *μ*g/ml HY concentration led to differentiation of a higher percentage of early apoptotic cells. Taken together, our results suggest that HY-PDT treatment triggered rapid cell death via apoptosis in HepG2 cells treated with 1 *μ*g/ml HY.

### HY-PDT treatment leads to release of reactive oxygen species (ROS) in HepG2 cells

**Figure 5:**
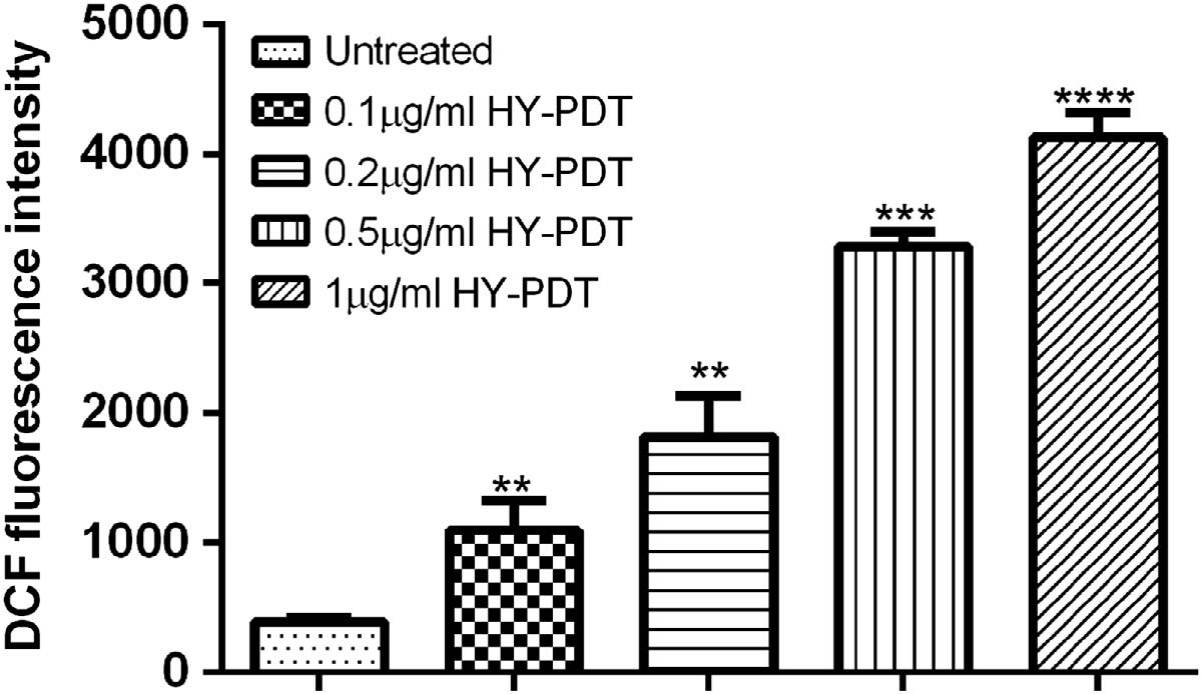
HY-PDT triggers ROS induction in HepG2. Intracellular ROS production was measured by oxidized dichlorofluorescin (DCF) levels in HepG2 cells exposed to increasing concentrations of HY and light irradiation. ROS measurement was performed 18 h after PDT treatment and increased level of ROS was observed in 1 *μ*g/ml HY-PDT-treated cells. Data are mean±S.E.M. of *n*=3 independent experiments carried out in triplicates. Statistically significant differences are labeled as *P<0.05, **P<0.01, ***P<0.001 and ****P<0.0001, compared with untreated cells using Student's unpaired two-tailed *t*-test

Next, we set out to determine whether ROS generation was involved in contributing to cell death after HY-PDT treatment, by measuring the fluorescence levels of 2′,7′-dichlorofluorescein (DCF) in the HY-PDT-treated cells. Our findings showed that lack of PDT treatment showed no significant increase in oxidized DCF levels. However, the amount of oxidized DCF was increased in the 0.1 and 0.2 *μ*g/ml HY-treated cells as compared with non-apoptotic cells (Figure 5). The fluorescence level of DCF was further increased in the 0.5 *μ*g/ml HY-treated cells and peaked at 1 *μ*g/ml HY-treated cells with irradiation. Taken together, these observations suggest that oxidization of DCF was higher in the 1 *μ*g/ml HY-treated cells, resulting in a high level of ROS production after PDT treatment (*P*<0.001). This effect was further validated by quantitative measurement of released *CYT-C* in HY-PDT-treated cells by quantitative real-time PCR (qRT-PCR; Figure 7).

### HY-PDT treatment leads to the upregulation of IL-6 in HepG2 cells

**Figure 6:**
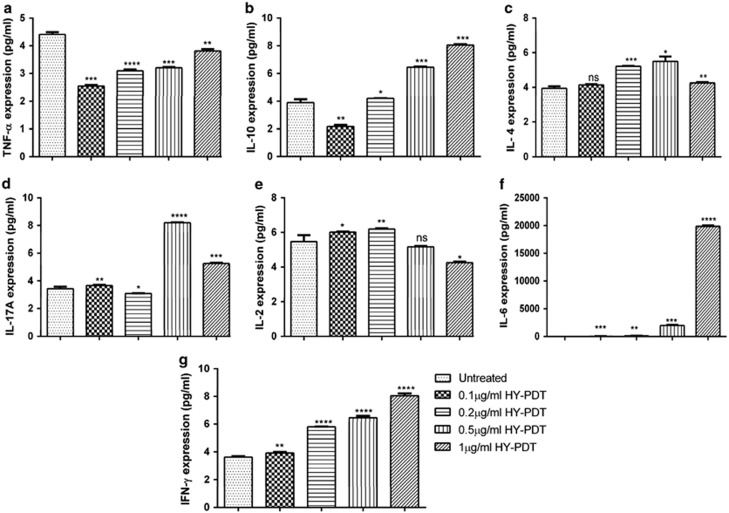
HY-PDT treatment triggers secretion of proinflammatory and anti-inflammatory cytokines in HepG2 cells. The cells were exposed to HY at increasing concentrations (0.1, 0.2, 0.5 and 1 *μ*g/ml) for 8 h followed by light irradiation for 5 min and 12 h post-incubation. The culture supernatant of each treated cells were analyzed for secretion of cytokines. Expression of TNF-*α* (**a**), IL-10 (**b**), IL-4 (**c**), IL-17A (**d**), IL-2 (**e**), IL-6 (**f**) and IFN-*γ* (**g**) were examined. Data are mean±S.E.M of *n*=3 independent experiments carried out in triplicates. Statistically significant differences are labeled as *P<0.05, **P<0.01, ***P<0.001 and ****P<0.0001, compared with untreated cells using Student's unpaired two-tailed *t*-test; ns, nonsignificant

To characterize the cytokine expression profile of HepG2 cells following *in vitro* PDT treatment, we determined the expression pattern of pro-inflammatory Th1 (IL-2, IL-6, TNF-*α* and IFN-*γ*), Th2 (IL-4), immunoregulatory (IL-10) and Th17 (IL-17A) cytokines in the culture supernatants. Figure 6 shows that HY-PDT strongly enhanced the expression IL-6 levels in the PDT-treated culture supernatants. The IL-6 levels were significantly increased following PDT with 0.5 *μ*g/ml HY (670 pg/ml; *P*<0.001) along with maximum stimulation with 1 *μ*g/ml HY (19 400 pg/ml; *P*<0.0001) after PDT as compared with HY treatment at 0.1 (13 pg/ml) and 0.2 *μ*g (59 pg/ml) with light irradiation. Stimulation of IL-10 (*P*<0.001), IL-4 (*P*<0.01) and IFN-*γ* (*P*<0.001) following PDT treatment occurred in a similar way, although in clearly lesser amounts as compared with IL-6. In parallel, IL-2 and IL-17A levels were all inconsistent after HY-PDT treatment and were mostly lying below the detection limit as compared with the other cytokines investigated. The concentration of other cytokines, with the exception of TNF-*α*, was increased after HY-PDT treatment as compared with the mock untreated cells. Hence, these results convincingly indicate that expression of IL-6 in the HY-PDT-treated cells was responsible for facilitating inflammation leading to tumor cell death.

### HY-PDT triggers cell death via recruitment of apoptotic caspase signaling in HepG2 cells

**Figure 7:**
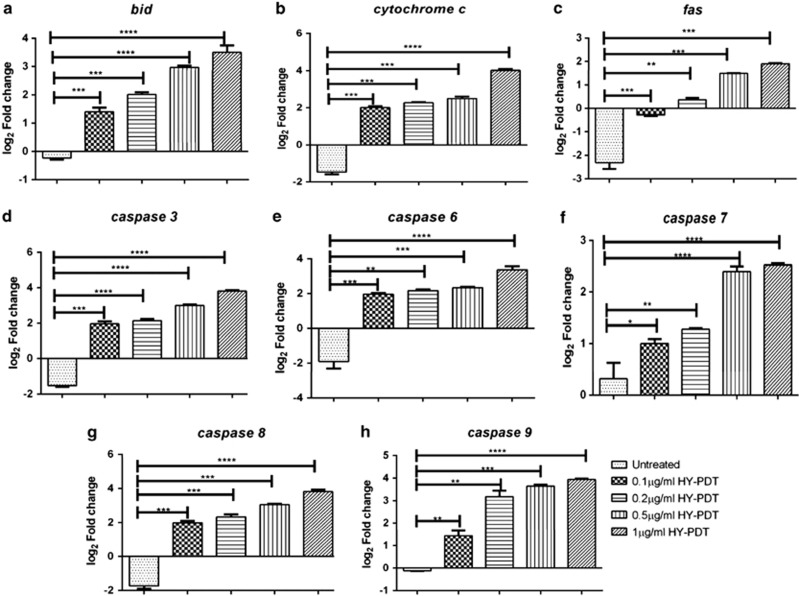
HY-PDT treatment induces expression of apoptotic mediators, *viz*., *BID, CYT-C, CASP3, CASP6, CASP7, CASP8, CASP9* and *FAS* in untreated and HY (0.1, 0.2, 0.5 and 1 *μ*g/ml)-treated HepG2 cells. The gene expression analysis was performed at 12 h post HY-PDT treatment. Genes were classified as upregulated relative to untreated cells if the expression was >0.5-fold or as downregulated if the expression was <0.5-fold, compared with untreated HepG2 cells. Log_2_-fold change of *BID* (**a**), *CYT-C* (**b**), *FAS* (**c**), *CASP3* (**d**), *CASP6* (**e**), *CASP7* (**f**), *CASP8* (**g**) and *CASP9* (**h**) were calculated with expression of *GAPDH* taken as internal control for normalization of real-time qRT-PCR data. Data are mean±S.E.M. of *n*=3 independent experiments carried out in triplicate. Statistically significant differences are labeled as *P<0.05, **P<0.01, ***P<0.001 and ****P<0.0001, compared with untreated cells using Student's unpaired two-tailed *t*-test; ns, nonsignificant

To investigate the molecular mechanism underlying the apoptotic pathway, we examined the expression levels of distinct caspases and pro-apoptotic genes. Expression of eight genes (Table 1) was assayed in HepG2 cells treated with different concentrations of HY and irradiation. The level of expression was normalized to untreated control cells and calibrated with reference to glyceraldehyde 3-phosphate dehydrogenase (GAPDH) expression. Upregulation of several apoptotic genes was observed in the irradiated HY-treated cells. Overall, the genes encoding *CYT-C* and *BID* were upregulated to 18-fold in all the treated cells. Meanwhile, the apoptotic caspases *CASP3, CASP6, CASP9* and *CASP8* were also upregulated to 10-fold in the HY-treated cells. We found that the apoptotic caspase *CASP7* was increased by 8.6-fold and continued to increase further with increasing concentrations of HY (Figure 7). Intriguingly, the death receptor *FAS* was downregulated in all the HY-treated samples except the 0.2, 0.5 and 1 *μ*g/ml HY-treated cells (Figure 7). We also observed increased expression of *CASP6* and *CASP9* in all the HY-PDT-treated cells. Collectively, these results indicate that increased expression of *CYT-C* is key to activation of apoptotic effectors *CASP3, CASP6* and *CASP7* to be able to induce cell death.

## Discussion

The success of current cancer therapeutics such as chemotherapy, cryosurgery, laser photocoagulation, radiation therapy and liver transplantation in treating HCC as well as other selected liver cancers continues to remain a critical issue for the broader application of treatment strategies in cancer.^1^ In addition, cancer metastasis occurring as a result of tumor progression reportedly facilitates an immune tolerant microenvironment and offsets cytotoxic immune and cellular responses in the adjacent areas of tumor especially during the late stages of cancer.^10^ Metastases can rarely be controlled by these treatments and, hence, complete resolution or cure remains scarce. Of late, therapeutics targeting the host immune system is increasingly being recognized as the most prominent approach to treatment of cancer.^13^ One such approach is the use of PDT as the preferred choice of cancer therapy in the twenty-first century, which has been proven to be a potent therapeutic approach for various cancers.^4^ The ability of PDT to specifically recognize and selectively destruct cancer cells while leaving the rest of the body's normal cells unharmed indeed is considered to be a milestone ground-breaking development.^9^

In general, PDT leads to release of ROS selectively by tumor cells, resulting in therapeutic stress inducing either necrosis (immediate cell death) or apoptosis (programmed cell death) of the tumor by the upregulation of inflammatory mediators (inflammation) while leaving the healthy tissues unaffected.^11^ It is widely believed that most of the PDT-mediated cell death is initiated in the mitochondria, ensuing activation of the mitochondrial pathway involving apoptotic mediators and caspases, the major determinants of apoptosis.^4^ In a patient with a competent immune system, it could lead to the release of tumor antigens or immunogens, which helps combat cancerous tumors at distance sites throughout the body.^16^ Hence, in this study, the interaction between proinflammatory cytokines in the tumor cell microenvironment and activation of apoptotic caspases in the presence of *CYT-C* and *BID* as pro-apoptotic factors was investigated in HepG2 cells following HY-PDT treatment.

Our study supports the observation that HY-PDT could facilitate multiple important events in tumor cell death.^17^ Notably, we have shown the ability of HY-PDT to inhibit tumor cell proliferation in a dose-dependent manner.^15^ We also showed that tumor cell-killing profile of irradiated HY-PDT was much stronger than that of non-irradiated HY, which is attributed to PDT treatment. These findings clearly show that tumor cell proliferation is mainly dependent on the intensity of light than the concentration of drug used. For instance, the IC_50_ value of HY was markedly decreased from 20 *μ*g/ml (data not shown) to 0.6 *μ*g/ml at 24 h after the PDT treatment in HepG2 cells (Figure 2). The high degree of cytotoxicity observed in 1 *μ*g/ml irradiated HY-treated HepG2 cells could be owing to alternations in the apoptotic mechanisms that reportedly render the cells more sensitive to HY-PDT.^18^ A recent study reviewed that HY has little noticeable biological activity in the absence of light and enhances the degree of undesirable side-effects on non-target tissues.^19^

The presence of annexin-FITC^+^/PI^−^ cell populations following HY-PDT treatment, in addition to cellular and nuclear damages as evident from DNA fragmentation experiments, and the formation of ROS are suggestive of recruitment of mechanisms consistent to apoptosis. We have convincingly established that irradiated cells with 1 *μ*g/ml HY treatment resulted in DNA laddering (Figure 3b). DNA fragmentation is reportedly suggestive of nuclear damage during the late stages of chromatin condensation.^20^ In contrast, DNA laddering was markedly lesser in the non-irradiated HY-treated cells (Figure 3a). We also showed phosphatidylserine externalization in the 1 *μ*g/ml HY-treated cells. Furthermore, we also showed that cells treated with 0.5 *μ*g/ml HY similarly showed phosphatidylserine externalization with signs of differentiation of early apoptotic cells. This is because phosphatidylserine externalization marks the early onset of apoptosis, and that cells treated with a lower dose of HY showed signs of early apoptosis but devoid of any signs of DNA fragmentation (that usually occurs during late apoptosis).^20^ Our findings on increased levels of ROS following HY-PDT fortify the association of HY-PDT with apoptosis.^21^ The ROS synthesis reportedly exceeds the intracellular antioxidant levels and induces macromolecular damage by interacting with cellular DNA, proteins and lipids leading to DNA fragmentation. Importantly, we also showed that a high level of ROS was produced during HY-PDT therapy, especially in cells treated with 1 *μ*g/ml HY. Here, we hypothesized that HY-PDT induced the secretion of ROS could have an important role in apoptotic signaling pathway. Others have shown that a low dose of PDT induces the release of endogenous ROS that could improve PDT toxicity.^22^ We also hypothesized that the photoactivated ROS in mitochondria would induce the release of CYT-C, which further activates pro-apoptotic caspases.^23^

Our experiments have clearly shown that HY-PDT leads to increased release of IL-6 in the tumor microenvironment. Recent literature on murine experiments has provided evidence that IL-6 could be upregulated following PDT directed at subcutaneous tumors.^24^ Investigations on human cervical carcinoma cells have provided evidence that PDT could increase IL-6 expression following activation of activator protein-1.^25^ Hence, our findings are in agreement with previously published findings that have convincingly established the increased presence of IL-6 in the tumor microenvironment following PDT.^13, 25, 26^ IL-6 serves as an inducer of chronic inflammation in addition to eliciting specific cellular immune responses to damaged cells, including T-cell activation.^27^ IL-6 is believed to be secreted at high doses than are conventionally used in radiation therapy, and the nature of cell death post radiation could be variable and could be owing to apoptosis, necrosis and/or autophagy. Immune-mediated cell death following radiation initiates the uptake of dead cells by dendritic cells, cross-presentation of the tumor-derived antigens to thymic lymphocytes (T cells) and eventual activation of tumor-specific T cells.^28^ It could also increase the permeability of the local vasculature through proinflammatory cytokine production leading to diapedesis of circulating inflammatory leukocytes to the site of inflammation. Further, PDT also reportedly induce certain heat-shock proteins, such as, heat-shock protein 70 and heat-shock protein 90, that facilitate the recruitment of tumor-specific cytolytic T cells.^29^ Our observations on increased IL-10 and IL-4 (albeit not significant) following HY-PDT probably explain the ability of the cytokines to potentially inhibit tumor development and progression.^29^ IL-10 reportedly inhibits development of regulatory T cells, a key component of immunosuppressive anti-tumor response.^30^ On the basis of these results, we propose a model in which upregulation of IL-6 in HepG2 cells following HY-PDT treatment activates tumor cell death via inflammation,^31^ and that secretion of IL-10 from HY-PDT-treated HepG2 cells alters tumor microenvironment promoting the suppression of tumor growth.^32, 33, 34, 35^

**Figure 8:**
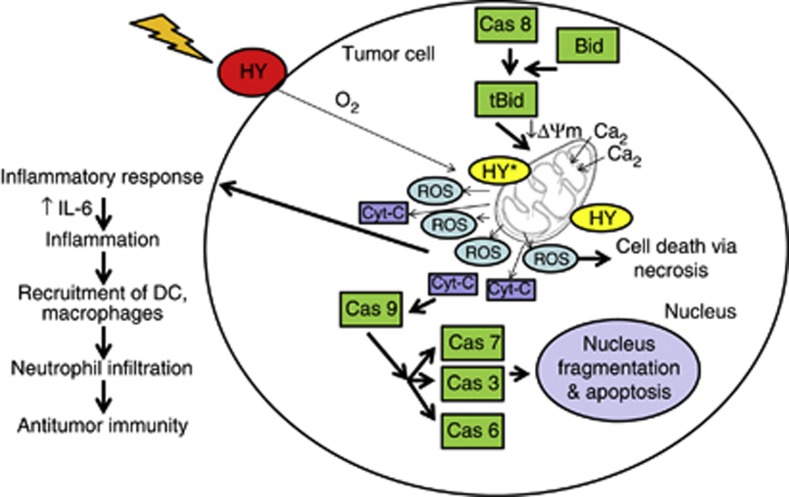
Proposed model of HY-PDT-induced cell death. We propose a mechanism of tumor cell killing by HY-PDT. The HY, lipophilic molecule, accumulates specifically in the tumor cell membrane^42^ and enters into the cells via endocytosis, pinocytosis or passive diffusion. The HY binds to mitochondria and become activated following irradiation with visible light. These activated HY react with O_2_ to produce cytotoxic reactive oxygen species (ROS), responsible for direct tumor cell killing. The HY-PDT results in rapid initiation of an inflammatory response eventually recruiting mediators of inflammation and stimulating tumor cells to release secondary inflammatory mediators for instance, IL-6, TNF-*α* and IFN-*γ* in treated cells. The treatment could also promote the accumulation of DC, macrophages and neutrophils leading to tumor destruction.^43^ Overall, HY-PDT is considered vital for the activation of antitumor immunity. On the other hand, induction of ROS also inclines tumor cell death via the mitochondrial apoptotic pathway. First, release of Ca^2+^ from the endoplasmic reticulum^43, 44^ and mitochondria, which has a major role in apoptosis followed by loss of mitochondrial membrane integrity and release of cytochrome c. The Bid cleavage coincided with translocation of tBid from cytoplasm to mitochondria and remarkably activates initiator caspases (caspase-8 or caspase-9) leads to the activation of effector caspases (caspase-3, -6 and -7). The prolonged oxidative stress resulting digestion of nucleosomal DNA into oligonucleosomes and visualize as DNA laddering at the late stage of apoptosis. Under certain circumstances, PDT-treated tumor cells could also undergo necrosis

Our investigations have clearly demonstrated that all HY-PDT-treated cells showed almost similar patterns of gene expressions, which include upregulation of apoptotic initiator (*CASP8* and *CASP9*), apoptotic effector caspase genes (*CASP3, CASP6, CASP7*) and pro-apoptotic genes (*BID* and *CYT-C*).^36^ HY-PDT-induced apoptosis usually operates via two main pathways (see Figure 8): the cytoplasmic pathway initiated by CASP8 activation, which subsequently triggers BID and downstream effector caspase activities,^28, 36, 37^ and the mitochondrial pathway, following light activation of HY, resulting in the loss of mitochondrial membrane integrity, eventually leading to the release of CYT-C from mitochondria into the cytosol.^23^ CYT-C potentially interacts with apoptotic protease-activating factor-1 through caspase recruitment domains to form apoptosome, which activates CASP9 and subsequently the activation of CASP3, CASP6 and CASP7.^36^ Importantly, we showed that CASP3 activity was increased sharply after CYT-C loss in line with previous findings.^37^ This suggests that these caspases could activate target molecules, nuclear lamins and actin, which are responsible for nuclear envelope disruption and apoptosis.^38^ Altogether, our current findings showed that HY-PDT induces changes in the tumor microenvironment by upregulating IL-6 causing chronic inflammation^39, 40^ and tumor growth inhibition, respectively, culminating in tumor cell death. There is concrete evidence to support our findings that upregulation of IL-6 following HY-PDT leads to increased apoptosis via apoptotic caspase functions.^24, 26, 33, 41^

In summary, this study establishes secretion of IL-6 by tumors cells following HY-PDT as a potential contributor of immune-stimulatory effects of apoptosis in tumor suppression. We propose that several distinct caspases functions through the apoptosis signaling pathways contributing to tumor suppression in HepG2 cells following HY-PDT treatment. Our observations suggest that HY-PDT induced apoptosis in HepG2 cells by facilitating increased IL-6 secretion and inflammation, to potentially recruit inflammatory cells, providing additional hints for the existence of alternative mechanisms of anti-tumor immunity in HCC.

## Materials and Methods

### Reagents

Pen-Strep solution (penicillin G 100 U/ml and streptomycin 100 mg/ml), ℒ-glutamine, Rosewell Park Memorial Institute1640 medium (RPMI 1640) medium without phenol red and heat-inactivated fetal bovine serum were purchased from Invitrogen (Paisley, UK). HY was purchased from Sigma-Aldrich (St. Louis, MO, USA).

### Cell lines and culture

Human HCC cell line, HepG2 (HB-8065), was purchased from American Type Culture Collection. The cells were cultured in complete RPMI 1640 medium supplemented with 10% (v/v) heat-inactivated fetal bovine serum, ℒ-glutamine (2 mM) and Pen-Strep solution at 37 °C with 5% CO_2_ in a humidified atmosphere. The cells were passaged every 3 days. HY was dissolved in sterile 0.1% (v/v) DMSO and stored at −20 °C. Dilutions were made using RPMI containing 0.1% (v/v) DMSO as final concentration.

### *In vitro* PDT treatment

PDT was performed on the HepG2 cells adhering to the protocols previously published in the literature.^6^ In brief, ∼1 × 10^6^ cells were seeded into each well of a flat-bottom six-well tissue culture plate containing 2 ml of complete RPMI 1640 medium. After 24 h of incubation, the medium in each well was removed and fresh medium containing various concentrations of HY (0, 0.1, 0.2, 0.5 and 1 *μ*g/ml) were added and further incubated for 8 h in the dark. Subsequently, the cells were exposed to halogen lamp irradiation for 5 min at 70 mW/cm^2^ light intensity and 593 nm wavelength. After the treatment, the cells were further incubated for 12 h in the dark. Later, the cells were examined (X100 magnification) using a Zeiss AX10 inverted phase-contrast microscope with epifluorescence and a digital imaging system (Carl Zeiss Microscopy GmbH, Gottingen, Germany). HepG2 cells treated in a similar manner but without light irradiation and/or HY were used as controls.

### Cell viability

The cell viability assay was conducted as previously described by others.^14^ In brief, ∼2 × 10^4^ cells were seeded into each well of a 96-well tissue culture plate a day before PDT treatment. The medium was aspirated and replaced with fresh medium containing 0.1% (v/v) DMSO and HY at doses ranging from 0 *μ*g to 1 *μ*g. Subsequently, the cells were incubated for 8 h and a specific light dose (70 mW/cm^2^ at a wavelength of 593 nm) was delivered to the cells followed by incubation for another 12 h. Following incubation, 10 *μ*l of PrestoBlue cell viability reagent (Life Technologies Inc., Van Allen Way, CA, USA), a resazurin-based solution, was added into each well and incubated further for 1 h. The fluorescence at 560 nm was recorded using a Varioskan Flash micro-plate reader (Thermo-Scientific, Erlangen, Germany). Cell viability was determined relative to the untreated cells.

### DNA fragmentation assay

Following HY-PDT treatment, the cells were harvested and centrifuged at 600 × *g* for 5 min at 4 °C. The resulting pellets were collected and the DNA was extracted using a commercial QIAamp DNA mini kit (Qiagen, Valencia, CA, USA) according to the manufacturer's instructions. The DNA quantity in each sample was measured and 10 *μ*g of the DNA were electrophoresed on 1.5% (w/v) agarose gel. Later, the gel was visualized using a UV light source and imaged using a Gel Doc XR gel documentation system (Bio-Rad, Hemel Hempstead, UK).

### Apoptosis assay

The PDT-treated and untreated cells were harvested and pelleted by centrifuging at 600 × *g* for 5 min at 4 °C. The intact cells (FITC^−^/PI^−^), early apoptotic cells (FITC^+^/PI^−^) and dead cells (FITC^+^/PI^+^) were acquired on a BD FACSCanto II (BD Biosciences, San Jose, CA, USA) flow cytometry using a BD Pharmingen FITC Annexin V Apoptosis Detection Kit I (BD Biosciences) according to the manufacturer's instructions. The samples were analyzed using the FACSDiva software (BD Biosciences).

### Determination of ROS

Determination of ROS levels was performed according to the manufacturer's instructions (Mitosciences, DCFDA Cellular ROS Detection Assay Kit; Mitoscience/Abcam, Cambridge, UK). The kit uses a cell permeant reagent 2′,7′-dichlorofluorescein diacetate (DCFDA), a fluorogenic dye that measures hydroxyl, peroxyl and any other intracellular ROS activities. Following diffusion into the cell, DCFDA is deacetylated by cellular esterases to a non-fluorescent compound, which is subsequently oxidized by intracellular ROS into DCF. DCF is highly fluorescent and could be detected using fluorescence spectroscopy at 495 nm (excitation) and 529 nm (emission). In brief, the cells were seeded overnight in a 96-well plate in 100 *μ*l culture medium in the dark. On the day of experiment, the cells were washed with 1 × phosphate buffered saline and subsequently incubated with 25 *μ*M DCFH-DA in the dark for 45 min. Later, DCFH-DA was removed, and the cells were washed with 1 × buffer solution followed by PDT treatment with different concentrations of HY. The fluorescence emanating from the cells of each well was measured and recorded at 485 nm (excitation) and 535 nm (emission) using a Varioskan Flash micro-plate reader (Thermo-Scientific).

### Fluorescence-activated cell sorter analysis

The supernatant from the PDT-treated and untreated cells were collected and filtered through a 0.22-*μ*m steriflip filter (Millipore, Billerica, MA, USA). The cytokines released from each sample were determined using a BD Cytometric Bead Array (CBA) Human Th1/Th2/Th17 Cytokine kit (BD Biosciences) according to the manufacturer's instructions. The assay was performed in triplicates and mock untreated cells were used as control. The assay detects IL-2, IFN-*γ*, TNF-*α*, IL-6, IL-10, IL-4 and IL-17A cytokines. The samples were acquired on a FACSCanto II (BD Biosciences) and analyzed using the FCAP array software (BD Biosciences).

### One-step qRT-PCR

Following PDT treatment, the total RNA of PDT-treated and PDT-untreated cells was extracted using a RNeasy Mini kit (Qiagen) as described in the manufacturer's protocol. One-step qRT-PCR amplification was performed using a QuantiTect SYBR Green RT-PCR kit (Qiagen) on a iQ5 Multicolor Real-Time PCR Detection System (Bio-Rad), and the results were analyzed using the iQ5 detection system. The amount of target gene expression levels was quantified using the formula 2^−ΔΔct^, where ΔΔ*C*_T_=(*C*_T,_ target−*C*_T,_ GAPDH) treated−(*C*_T_, target−*C*_T,_ *GAPDH*) untreated. The gene expression level was normalized to GAPDH. Primer sets for human *β-tubulin, BID, CYT-C, CASP3, CASP6, CASP7, CASP8, CASP9, GAPDH* and *FAS* were obtained from the Primer Bank (http://pga.mgh.harvard.edu/primerbank/; Table 1). The primers used in the investigation were synthesized by First Base Laboratories (First Base, Kuala Lumpur, Malaysia).

### Statistical analysis

Data are presented as the mean±S.E.M. and *n* refers to the number of independent experiments. Levels of significance for comparisons between two or more independent samples were determined using a two-tailed unpaired Student's *t*-test. Differences were considered significant at *P*<0.05. Groups were compared by one-way or two-way analysis of variance with Bonferroni's *post hoc* test applied to explore significance.

## Figures and Tables

**Table 1:** List of quantitative real-time PCR templates with forward and reverse primers

**Template**	**Forward primer (5′–3′)**	**Reverse primer (5′–3′)**
Caspase 3	AGAGGGGATCGTTGTAGAAGTC	ACAGTCCAGTTCTGTAACCACG
Caspase 8	CACCTTGTGTCTGAGCTGGTCTG	CACCCAGGGGCTGCTCCTTCT
Caspase 9	CACTTCCCCTGAAGACGAGTC	GTGGGCAAACTAGATATGGCG
Caspase 6	ATGGCGAAGGCAAATCACATTT	GTGCTGGTTTCCCCGACAT
Caspase 7	GGGACCGAGTGCCTACATAT	CGCCCATACCTGTCACTTTATCA
BID	CCTTGCTCCGTGATGTCTTTC	TAGGTGCGTAGGTTCTGGT
CYT-C	AGTGTTCCCAGTGCCACACCG	TCCTCTCCCCAGAATGATGCCTTTG
FAS	GAGTTTCTGTTCGGACTGTGC	TGTTACAGGAAAGCTGATCTGTC
GAPDH	AAGGTGAAGGTCGGAGTCAAC	GGGGTCATTGATGGCAACAATA
Tubulin	AACACGGGATCGACTTGGC	CTCGGGGCACATATTTCCTAC

## Abbreviations

BID: BH3-interacting-domain death agonist
CARD: caspase recruitment domain
CASP: caspase
CYT-C: cytochrome complex
DC: dendritic cell
DCF: 2′,7′-dichlorofluorescein
DCFDA: 2′,7′-dichlorofluorescein diacetate
FAS: Fas (TNF receptor superfamily member 6)
FITC: fluorescein isothiocyanate
GAPDH: glyceraldehyde 3-phosphate dehydrogenase
HCC: hepatocellular carcinoma
HepG2: human hepatocellular liver carcinoma cell line
HeLa: human cervical carcinoma
HY: hypericin
IFN-*γ*: interferon-*γ*
IL: interleukin
PDT: photodynamic therapy
PS: photosensitizer
PI: propidium iodide
qRT-PCR: quantitative real-time PCR
ROS: reactive oxygen species
RPMI 1640: Rosewell Park Memorial Institute1640 medium
T cells: thymic lymphocytes
TNF-*α*: tumor necrosis factor alpha
